# Kimura’s disease: A clinicopathological study of 23 cases

**DOI:** 10.3389/fmed.2022.1069102

**Published:** 2022-12-29

**Authors:** Chih-Chun Lee, Kuang-Hui Yu, Tien-Ming Chan

**Affiliations:** ^1^Department of Medical Education, Chang Gung Memorial Hospital, Keelung, Taiwan; ^2^Division of Rheumatology, Allergy, and Immunology, Department of Internal Medicine, Chang Gung Memorial Hospital, Chang Gung University, Taoyuan, Taiwan

**Keywords:** Kimura’s disease, pathology, IgG4-related disease, Taiwan, eosinophilia

## Abstract

**Introduction:**

Kimura’s disease (KD) is an uncommon lymphoproliferative fibroinflammatory disorder. Patients present with head and neck subcutaneous nodules with or without lymphadenopathy. Peripheral blood eosinophilia and elevated serum immunoglobulin E (IgE) levels are typical. This study was designed to delineate the clinicopathological features, pattern of care, and disease course of 23 Taiwanese patients with KD.

**Methods:**

We retrospectively analyzed the clinical data of 23 consecutive cases (16 male and 7 female; age at diagnosis: 12–77 years) of KD diagnosed at our institution from 2015 to 2020.

**Results:**

The median time from presentation to diagnosis was 1 month. Twenty-one patients presented with unilateral or bilateral head and neck masses. The remaining two presented with right flank and right arm lesions, respectively. Peripheral blood eosinophilia was observed in nine, and elevated IgE levels were observed in four. All were diagnosed using either excisional or core-needle biopsy. Seven patients underwent fine needle aspiration without a diagnostic yield. Salivary gland and lymph node involvement was observed in three and seven patients, respectively. Most lesions showed tissue eosinophilia (100%) and florid follicular hyperplasia (78.26%). Three cases had histological KD-IgG4-RD overlap and three had comorbid IgG4-RD were recognized. Thirteen patients underwent surgical resection, one received adjuvant therapy, and two received prednisolone monotherapy.

**Conclusion:**

KD should be considered in patients with subcutaneous masses, eosinophilia, and elevated IgE levels. Biopsy remains the gold standard of diagnosis. Increased recruitment of IgG4^+^ plasma cells is a common feature. Consideration of IgG4-RD in all KD patients may be prudent.

## 1 Introduction

Kimura’s disease (KD) is an uncommon lymphoproliferative fibroinflammatory disorder which classically affects middle-aged Asian men, with a typical presentation of painless head and neck subcutaneous nodules, regional lymphadenopathy, peripheral blood eosinophilia, and elevated serum immunoglobulin E (IgE) levels. A history of atopy is frequently identified. The disease was first described by Kimm and Szeto in 1937 and better characterized by Kimura et al. in 1948 (1, 2).

The etiology of KD remains unclear. Studies have suggested that immunological dysregulation involving IgE-mediated type-1 hypersensitivity and T helper (Th) 2 cytokines plays a significant role in its pathogenesis, in which tissue injuries, allergies, infections, endocrine disorders, and autoimmunity are potential triggers (3–7). Studies have uncovered a pivotal role of Th 2 cytokines in the pathogenesis of KD. Elevated levels of interleukin (IL)-4, IL-5, and IL-13 mRNA expression have been reported in the peripheral blood mononuclear cells of patients with KD (8). Immunohistochemical examination of KD tissues further revealed increased infiltration of IL-4^+^ and IL-5^+^ mast cells and T cells along with increased infiltration of eotaxin^+^ and C–C motif ligand 5 [CCL5; also known as regulated upon activation, normal T cell expressed and presumably secreted, (RANTES)] + mast cells, T cells, and activated eosinophils (9), confirming the dominance of Th2 response.

The reactive nature of this disease is reflected in the distinct histopathologic patterns of florid follicular hyperplasia, inter- and intra-follicular dense eosinophilic and lymphoplasmacytic infiltration, eosinophilic micro-abscesses (with or without Charcot-Leyden crystals), increased small blood vessels, and prominent stromal fibrosis. Lymphoproliferation is associated with the proliferation of post-capillary venules. However, high endothelial venules are atypical. The replacement of normal germinal centers with IgE or eosinophilic deposits, necrosis, and polykaryocytes (Warthin–Finkeldey-type giant cells) can be present (4, 10). In some patients, inflammatory infiltration of nerve fibers results in skin irritation and pruritus (11).

Visceral involvement is a rare complication and can present late in the disease course, warranting a long-term follow-up. Renal complications are among the most documented, ranging from urinalysis abnormalities to renal insufficiency. Several pathologies have been associated with KD, including focal and segmental glomerulosclerosis, membranous glomerulonephritis, membranoproliferative glomerulonephritis, eosinophilic tubulointerstitial nephritis, minimal change disease, and acute tubular necrosis (12–15).

To characterize the clinicopathological features, pattern of care, and disease course of KD in Taiwanese patients. Data from 23 affected patients were retrospectively reviewed.

## 2 Materials and methods

In this retrospective single-institution multicenter study, patients diagnosed with KD from 2015 to 2020 at Chang Gung Memorial Hospital (CGMH), a tertiary referral medical institution in Taiwan including two medical centers with four main branches and six subsidiary establishments across the country, were analyzed. Medical charts of all the cases were collected from the time of diagnosis to September 2022. Clinical data before the diagnosis of KD were retrospectively examined to determine the time from initial presentation to disease diagnosis.

Kimura’s disease was defined pathologically, after the exclusion of granulomas and evidence of malignancy, with a constellation of tissue eosinophilia and eosinophilic microabscesses, florid follicular hyperplasia with prominent germinal centers, interstitial fibrosis, lymphoplasmacytic infiltrates, increased small blood vessels, and post-capillary venule proliferation.

The diagnosis of IgG4-related disease (IgG4-RD) was made according to the 2019 American College of Rheumatology (ACR)/European League Against Rheumatism (EULAR) classification criteria for IgG4-RD.

A detailed review of the medical records of the 23 consecutive cases, including laboratory, imaging, and pathology data, was conducted for the 23 consecutive cases.

This study was approved by the Institutional Review Board of Chang Gung Memorial Hospital (202100959B0). There was no need to obtain consent.

## 3 Results

### 3.1 Clinical characteristics

The clinical characteristics of the 23 cases were listed in Tables 1, 2.

**TABLE 1:** Clinical details of the 23 cases.

No.	Age/Sex	Duration (months)	Size of tumor mass (cm)	Max diameter (cm)	Volume (cm^3^)	Eosinophil (% of total white blood cells)	IgE (IU/dL)	Treatment	Comorbidities
KD001	36/M		1.4 × 0.7 × 0.2	1.4	0.196	5.8	193	Ex	Allergic urticaria
KD002	27/M	12	2 × 1 × 0.5	2	1	3.1	248	Ex	—
KD003	42/M		4.9 × 3.1 × 1.9	4.9	28.861	19.3	NR	Ex	—
KD004	41/M	1	1.3 × 0.1 × 0.1	1.3	0.013	11.9	NR	Ex	—
KD005	59/M		1 × 0.1 × 0.1	1	0.01	41	NR	—	Systemic mastocytosis and polycythemia vera
KD006	26/M	20	0.9 × 0.8 × 0.6	0.9	0.432	11.9	NR	—	—
KD007	39/F	1	0.4 × 0.4 × 0.1	0.4	0.016	4.1	3410	—	—
KD008	41/F	120	4.3 × 3.7 × 3	4.3	47.73	26	4090	Ex	Atopic dermatitis, allergic rhinitis
KD009	14/M	1	2.6 × 1.7 × 1	2.6	4.42	5.8	NR	Ex	Asthma, thalassemia minor
KD010	26/M	60	1.7 × 0.1 × 0.1	1.7	0.017	NR	NR	PSL	—
KD011	61/F	2	1.3 × 0.1 × 0.1	1.3	0.013	3.2	NR	PSL	IgG4-RD, allergic rhinitis, ANA(+) AC-3 centromere 1:1280
KD012	37/M		0.5 × 0.1 × 0.05	0.5	0.0025	4.5	NR	Ex, PSL	IgG4-RD
KD013	44/F	1	0.7 × 0.1 × 0.05	0.7	0.0035	6.5	NR	—	—
KD014	21/M	120	1.4 × 0.1 × 0.1	1.4	0.014	NR	NR	—	—
KD015	77/M	6	0.9 × 0.4 × 1.1	1.1	0.396	NR	NR	—	—
KD016	12/F	2	1.2 × 0.9 × 0.4	1.2	0.432	2	NR	Ex	—
KD017	46/M	61	2.3 × 1.4 × 1	2.3	3.22	NR	NR	Ex	—
KD018	55/M	24	0.7 × 0.25 × 0.5	0.7	0.0875	NR	NR	—	—
KD019	60/F	1	1.4 × 0.9 × 0.8	0.4	1.008	5	NR	Ex	Rheumatoid arthritis
KD020	35/F	1	1.8 × 1.6 × 0.7	1.8	2.016	NR	NR	Ex	—
KD021	54/M		1.1 × 0.4 × 0.8	1.1	0.352	2.7	NR	Ex	Eczema, pompholyx, urticaria, leukocytoclastic vasculitis, seborrheic dermatitis
KD022	18/M	1	0.45 × 0.1 × 0.1	0.45	0.045	NR	NR	—	—
KD023	30/M	1	2.4 × 2.1 × 0.5	2.4	2.52	22	NR	Ex	—

M, male; F, female; NR, not reported; Ex, excision; PSL, prednisolone; —, absent.

**TABLE 2:** Clinical features of the 23 cases.

Features of the 23 patients with Kimura’s disease	No. of patients (%)	*P*-value
**Age (years)**		
Median (QI, Q3)	39 (27, 50)	
<30	7 (30.43)	<0.01
≥30	16 (69.57)	
**Sex**		
Male	16 (69.57)	<0.01
Female	7 (30.43)	
**Laterality, *n* = 21**		
Unilateral	19 (90.48)	<0.001
Bilateral	2 (9.52)	
**Modalities of treatment, *n* = 14**		
Surgical excision	12 (85.71)	<0.01
Surgical excision only	11	
Surgical + steroids	1	
Steroid monotherapy	2 (14.29)	
**Anatomical site**		
Head and neck	21 (91.3)	<0.001
Non-head and neck	2 (8.7)	
Extremities	1	
Trunk	1	
**Tumor diameter (cm)**		
Median (QI, Q3)	1.3 (0.8, 1.9)	<0.01
<3	18 (78.26)	
≥3	5 (21.74)	
**Duration of symptom (years), *n* = 19**		
Median (QI, Q3)	0.08 (0.25, 1.92)	<0.01
<1	12 (63.16)	
≥1	7 (36.84)	

Of the 23 cases, 16 were male and seven were female. The age at diagnosis ranged from 12 to 77 years (median: 39 years).

Five patients had a history of atopy (i.e., atopic dermatitis, allergic rhinitis, asthma, and/or allergic urticaria); one of whom had concurrent eczema, pompholyx, urticaria, seborrheic dermatitis, and leukocytoclastic vasculitis. One patient had rheumatoid arthritis, one had IgG4-RD with positive anti-nuclear antibody (ANA) (AC-3 centromere 1:1,280), and one had systemic mastocytosis and polycythemia vera (systemic mastocytosis with an associated clonal hematological non-mast cell lineage disease) with JAK2V617F mutation and myelofibrosis. Two additional cases of IgG4-RD were recognized during the workup for KD.

**FIGURE 1:**
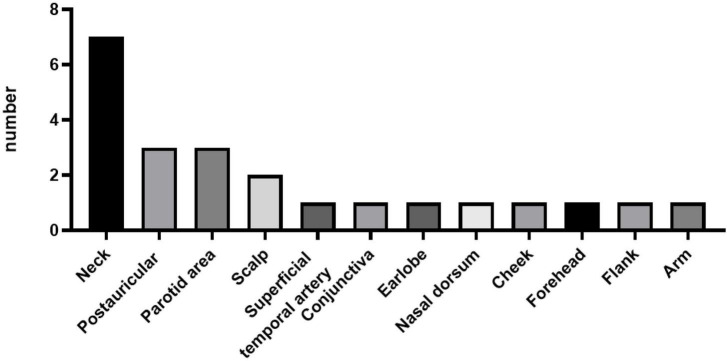
Distribution of Kimura’s disease lesions in various parts of the body of 23 patients.

**FIGURE 2:**
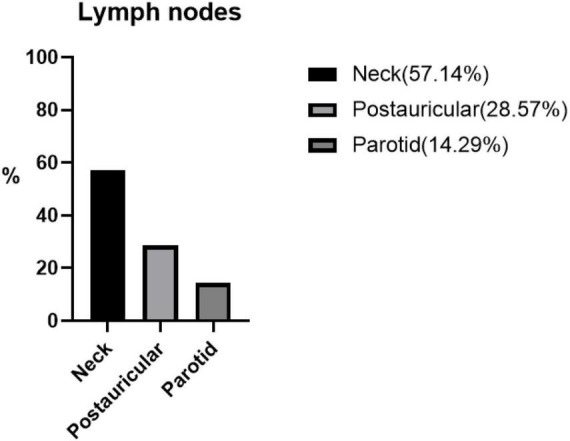
The percentage distribution of different lymphatic systems in seven patients with Kimura’s disease and lymphadenopathy.

Of the 23 patients, 21 patients presented with either unilateral (90.48%) or bilateral (9.52%) head and neck masses. The remaining two patients presented with right flank and right arm lesions, respectively. The detailed sites of involvement are shown in Figures 1, 2.

The maximal lesion diameter ranged from 0.4 to 4.9 cm.

### 3.2 Laboratory results

An increased proportion of eosinophils was observed in nine (56.25%) patients, ranging from 5.8% to 41%.

Four (100%) patients had elevated IgE levels, ranging from 193 to 4,090 IU/dL (normal range: 100 IU/dL).

### 3.3 Pattern of care and diagnostic modalities

Among the 23 patients who sought care from our institution, eight visited the Otolaryngology Department, five visited the Dermatology Department, three visited the General Surgery Department, and two visited the Pediatric General Surgery Department. The remaining five patients visited the Neurosurgery, General medicine, Ophthalmology, Plastic Surgery, and Infection departments, respectively.

All patients were definitively diagnosed with KD using either excisional or core-needle biopsy. Seven patients underwent fine needle aspiration of no diagnostic yield during initial workup. The time from presentation to diagnosis ranged from 1 to 120 months, with a median duration of 1 month.

Kimura’s disease was diagnosed by the microscopic identification of characteristic features after exclusion of granulomas and evidence of malignancy. Immunochemical staining was performed for some patients as a confirmatory measure.

Patients received treatment at the General Surgery (*n* = 3), Otolaryngology (*n* = 2), Plastic Surgery (*n* = 2), Dermatology (*n* = 2), Pediatric General Surgery (*n* = 2), Rheumatology (*n* = 2), and Neurosurgery (*n* = 1) departments.

Patients received follow-up at the Dermatology (*n* = 4), Rheumatology (*n* = 4), General Surgery (*n* = 3), Otolaryngology (*n* = 3), Plastic Surgery (*n* = 2), Hematology (*n* = 2), Pediatric Hematology (*n* = 2), Neurosurgery (*n* = 1), Ophthalmology (*n* = 1), and Infection (*n* = 1) departments. Mean follow up duration was 68.0 months (21–95 months).

### 3.4 Histopathological findings

**FIGURE 3:**
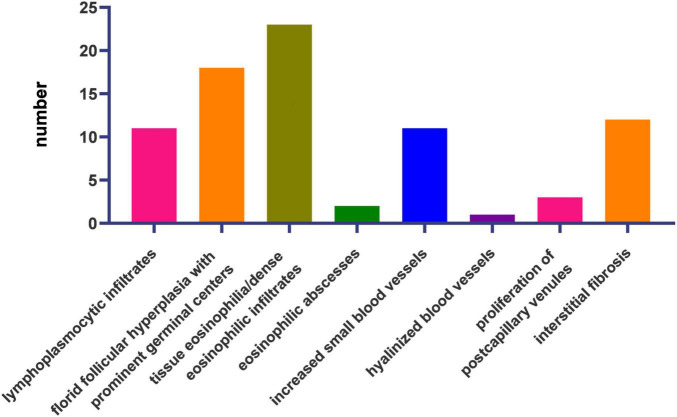
Histopathological features of the 23 cases.

Microscopic observation using hematoxylin and eosin staining was performed for all patients (Figure 3).

Lymph node involvement was in seven (30.43%) patients. Three (13.04%) patients had lesions involving the salivary glands.

The most frequent histological features were tissue eosinophilia (*n* = 23, 100%), florid follicular hyperplasia with prominent germinal centers (*n* = 18, 78.26%), interstitial fibrosis (*n* = 12, 52.17%), lymphoplasmacytic infiltrates (*n* = 11, 48.82%), and increased small blood vessels (*n* = 11, 48.82%). Proliferation of post-capillary venules (*n* = 4), eosinophilic microabscesses (*n* = 2), and hyalinized vessels (*n* = 1) were also present (Figure 3).

Immunochemical staining was performed in nine patients, revealing that three patients had overlapping histological features of IgG4-RD with IgG4 + /IgG + cell ratio > 40%.

### 3.5 Management

Of the 23 patients, 15 (65%) received treatment. Most patients (*n* = 12) underwent surgical resection. One patient received adjuvant prednisolone therapy with *per os* [po] prednisolone 10 milligrams [mg]/day for 5 months followed by 15 mg/day for another 7 months. Two patients received prednisolone monotherapy. One of them received oral prednisolone 15 mg/day for 17 months followed by 25 mg/day for 25 months and 20 mg/day for another 28 days. He subsequently lost follow-up. The other one received oral prednisolone 20 mg/day for 31 months followed by 10 mg/day for 36 months. She was still on a maintenance dose of 2.5 mg/day at the end of our study period.

## 4 Discussion

We presented a retrospective clinicopathological analysis of 23 cases of KD. Our results were compatible with those of previous reports regarding male predominance, a disease predilection for head and neck soft tissue, lymph nodes and salivary glands, and an association with allergic and autoimmune disorders. Furthermore, our report identified features of IgG4-RD as significant confounders in KD lesions, potentially warranting workup for IgG4-RD in all patients with KD. Furthermore, we demonstrated a widely varied duration from the initial presentation to diagnosis, reflecting the non-specific symptoms that lead to a heterogeneous pattern of care.

### 4.1 Gender differences in KD

According to a recent review of 238 patients, the male-to-female ratio in patients with KD was approximately 4:1 with more pronounced male predominance (17:1) in a younger population (16). In our series, the overall male-to-female ratio was 16:7. When stratified according to age, the ratio was 6:1 for those aged <30 years and 5:3 for those aged ≥30 years. The average percentage of eosinophils was 12.8% (2.7–41%) in males and 7.8% (2–26%) in females. The average lesion diameter was 1.55 cm (0.45–4.9 cm) in males and 1.44 cm (0.4–4.3 cm) in females. Salivary gland involvement tended to affect males (3/16 versus 0/7) while lymph nodes were more involved in females (3/7 versus 4/16). A dense lymphoplasmacytic infiltrate, increased small blood vessels and interstitial fibrosis were more prominent features in male KD patients (9/16 vs. 2/7, 9/16 vs. 2/7, and 10/16 vs. 2/7, respectively). Three men had features of IgG4-RD in their tissue samples. Female patients were more likely to present with atypical features of high endothelial venules (2/7 versus 2/16).

### 4.2 Differential diagnosis

One case (a 77-year-old male; KD015) in our series presented with a right flank mass. In contrast to the typical subcutaneous site of involvement, biopsy revealed an unusual dermal distribution of the typical features of KD, including marked tissue eosinophilia with follicular proliferation, spanning the entire dermis extending to the superficial subcutis. The pathologist characterized the lesion as a cutaneous counterpart of KD by the pathologist.

Another atypical case (a 55-year-old male; KD018) presented with a left malar subcutaneous lesion. The histological features, although more suggestive of KD, were consistent with eosinophilic (lobular) panniculitis. There has been one report on the coexistence of KD and eosinophilic panniculitis in the literature (17). The significance of this finding remains unknown. We suggest that being a non-specific tissue reaction pattern, eosinophilic panniculitis is better characterized as a possible associated finding in active KD lesions, with limited value in the final diagnosis.

Historically, angiolymphoid hyperplasia with eosinophilia (ALHE) was considered a vasculoproliferative counterpart of KD. The entity, typically affecting an older population with a more equal sex ratio, is distinguished histologically by profuse vascular proliferation with plump histiocytoid endothelial cells, scar lymphoproliferation and eosinophilic infiltrates, and cutaneous involvement (4). Peripheral blood eosinophilia and elevated IgE levels are rare, as well as lymphadenopathy. In our series, high endothelial venules were observed in four patients (a 26-year-old male, a 60-year-old female, a 35-year-old female, and a 54-year-old male; KD010, KD019, KD020, and KD021), rendering ALHE a potential differential diagnosis. The samples exhibited exuberant lymphoproliferation and abundant eosinophilic infiltrates, nevertheless, favoring the pathological diagnosis of KD.

### 4.3 Association with IgG4-RD

IgG4-RD is a distinct fibroinflammatory condition often manifested as tumefactive lesions, characteristically featuring a dense lymphoplasmacytic infiltrate with increased IgG4^+^ plasma cells, storiform fibrosis, obliterative phlebitis, and a mild-to-moderate degree of tissue eosinophilia. Follicular hyperplasia, a prominent feature of KD, has been described in IgG4-RD. Notably, marked tissue eosinophilia does not exclude the diagnosis. Since IgG4-RD is a clinicopathologically defined entity, the associated pathology, although highly consistent across involved organs, is not diagnostic in and of itself. Nevertheless, the constellation of features, as well as a similar tendency to cluster with allergic conditions (sometimes with peripheral blood eosinophilia and elevated serum IgE levels that may be associated with atopy), render IgG4-RD an important differential diagnosis of KD. Careful clinicoseropathological evaluation is crucial for pertinent diagnosis and optimal management.

In our series, increased recruitment of IgG4^+^ plasma cells, to a degree of an IgG4^+^/IgG^+^ cell ratio of >40%, was found in the otherwise typical KD lesions of three patients. Of whom two (a 42-year-old male and a 37-year-old male; KD003 and KD012) fulfilled the 2019 ACR/EULAR classification criteria for IgG4-RD (18). The remaining one patient (a 36-year-old male; KD001) had characteristic IgG4-RD lesion involving the right post-auricular lymph node without extranodular evidence of the disease, precluding the diagnosis of IgG4-RD. There was another tissue sample (of a 12-year-old female; KD016) displaying storiform fibrosis without increase in the percentage of IgG4 + plasma cells while none of the 23 patients possessed lesions with obliterative phlebitis.

Additionally, we found one case (a 61-year-old female; KD011) of recurrent left submandibular mass and lymphadenopathy, who underwent multiple biopsies in 2014, 2015, and 2017. Before 2015, her lesions were consistent with KD without evidence of increased IgG4^+^ plasma cells. However, in 2017, the recurred mass was classified as IgG4-RD with an IgG4^+^/IgG^+^ cell ratio of >40%. Elevated serum IgG4 levels were subsequently found during further rheumatological workup, as well as positive ANA of the centromere pattern (AC-3) with a titer of 1:1,280.

**FIGURE 4:**
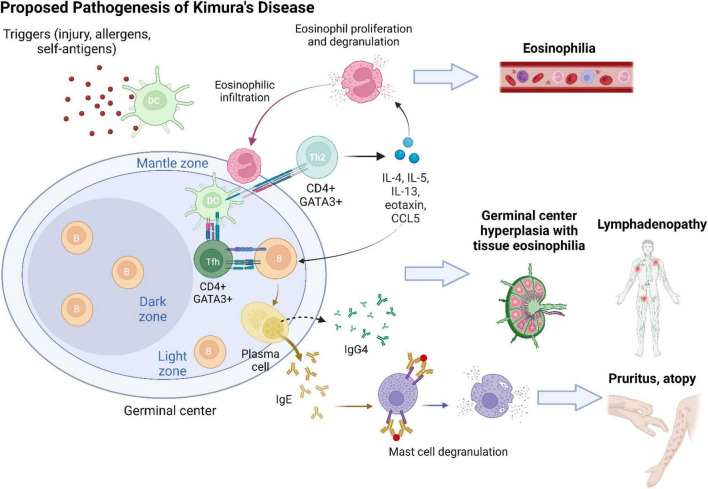
The development of Kimura’s disease is preceded by a series of chronic Th2-predominant inflammation. An inciting event (e.g., tissue injury and viral infection) leads to tissue exposure to self-antigens or entry of allergens. Antigens/allergens are taken up by dendritic cells (DCs) and presented to naive CD4 + T cells in germinal centers. Upon interaction with DCs, naive CD4 + T cells differentiate into CD4 + GATA3 + follicular T (Tfh) cells and helper T (Th2) cells. Tfh cells facilitate B cell class switching into IgE- and IgG4-secreting plasma cells. Antigen presentation *via* IgE activates mast cells and recruits them to KD lesion sites. Mast cell degranulation contributes to clinical symptoms of pruritus and atopy. Cytokines secreted by Th2 cells, including IL-4, IL-5, IL-13, eotaxin, and RANTES (regulated upon activation, normal T cell expressed and presumably secreted; CCL5), sustain a type 2 inflammatory milieu and induce activation and proliferation of eosinophils. Tissue infiltration of eosinophils and generalized eosinophilia ensue. Chronic antigen stimulation results in reactive lymphoproliferation with hyperplasia of germinal centers.

Kimura’s disease lesions are characterized by a chronic Th2 inflammatory milieu (Figure 4). T cell-derived IL-4 and IL-13 are separately known to induce B cell synthesis of IgE and IgG4 (19, 20). Infiltration of IgG4 + plasma cells has been variably reported in previous KD case series (5, 10, 21, 22). It has been postulated that as KD lesions progress to chronic stage, Th2 inflammation would be suppressed with resolution of type I hypersensitivity, resulting in an apparent increased proportion of IgG4 compared with IgE (22). While the predominant source of IgG4 in KD is presumably Th2 cells (23), it was recently revealed that a subset of profibrotic CD4 + cytotoxic T cells are the major instigators of IgG4-RD (24). Most recently, while investigating the nature of follicular T (Tfh) cells, researchers found two disease-specific conditions associated with distinct subsets of Tfh cells (25). It has been suggested that KD represents a process of “allergic fibrosis” that is Th2-driven, whereas IgG4-RD involves a form of “inflammatory fibrosis” orchestrated by cytotoxic T cells (25).

Without accounting for the genesis of IgG4, the 2019 ACR/EULAR classification criteria for IgG4-RD are inadept to discriminate between KD and IgG4-RD due to their phenotypic similarities. Another pitfall is the high comorbid rate of atopic conditions, a feature that may prohibit precise determination of the significance of an observed Th2 response in either disease. Moreover, despite the potential difference in the pathogenesis of KD and IgG4-RD, the presence of KD does not preclude IgG4-RD, and vice versa. Hence, the diagnosis and management of apparent KD-IgG4-RD overlap remain challenging. As increased IgG4 secretion is likely an epiphenomenon in both conditions, future investigations comparing the two conditions may shed more light on their respective pathogenesis, as well as the implications of elevated IgG4 in disease manifestation and progression.

Since malignancies are known complications of IgG4-RD, our finding of high prevalence of IgG4-RD in patients with KD suggests that workup for IgG4-RD—immunochemical staining of lesion samples along with serological testing for IgG4 levels—is prudent in all patients with or under suspicion of KD. In light of our current understanding of their respective natural history, aggressive corticosteroid therapy with an emphasis on renal function monitoring is likely warranted for the management of these cases of KD-IgG4-RD overlap. Further studies comparing the clinical course and outcomes of KD, KD/IgG4-RD, and IgG4-RD are needed to provide more clinical guidance on patient risk stratification and optimal therapeutics.

### 4.4 Visceral involvement

None of the 23 patients enrolled in this study had documented visceral involvement. This may reflect the rarity of such complications; the detection of which is further limited by the short follow-up duration. Long-term follow-up with urinalysis and guided by clinical clues may be warranted to detect potential late-onset visceral involvement, particularly for those who received local management (surgical resection) alone or no treatment.

### 4.5 Selection of therapy and outcomes

A treatment algorithm for KD was recently proposed. Patients were stratified according to lesion size, disease duration, and laboratory results. The mainstay treatment consisted of surgical resection. Radiation therapy and immunosuppressive agents are considered add-ons as a part of salvage or adjuvant therapy to prevent recurrence in high-risk patients (26).

Surgical resection was the major treatment modality in our series. The incorporation of prednisolone was deliberated based on the presence of features of IgG4-RD and the degree of eosinophilia.

Of the two patients newly diagnosed IgG4-RD, one underwent surgical resection and subsequent prednisolone therapy at the Rheumatology Department, whereas the other patient underwent surgical resection alone at the Otolaryngology Department. As noted previously, further studies on the management of KD versus KD/IgG4-RD phenotypes are of interest.

Two patients received prednisolone monotherapy. One presented with antecedent diagnosis of IgG4-RD and the other had marked eosinophilia (41%); both of whom were managed by rheumatologists.

Apart from the one case with recurrent left submandibular mass, no short-term treatment complications or recurrence were noted in this series. The relatively short follow-up time precludes the detection of long-term outcomes.

## 5 Conclusion

Kimura’s disease should be considered in patients with subcutaneous masses, peripheral eosinophilia, and elevated IgE levels with or without a history of atopy. Biopsy remains the gold standard of diagnosis, yielding better results. Increased recruitment of IgG4 + plasma cells, sometimes fulfilling the diagnosis of IgG4-RD, is not an uncommon feature, complicating the diagnosis and management, and possibly warranting workup for IgG4-RD in all patients under the evaluation for KD. As increased IgG4 secretion appears to be an epiphenomenon in both KD and IgG4-RD, the current ACR/EULAR classification criteria for IgG4-RD may not be adept to differentiate the two conditions. Future endeavors in comparing the two conditions may shed more light on their respective pathogenesis and the clinical implications of elevated IgG4. Potential visceral involvement appears to be a largely overlooked aspect of KD in clinical practice. Larger series with longer follow-up time are needed to further demonstrate the prognosis of KD in Taiwanese patients. Particularly of interest are the rates of visceral involvement and recurrence, as well as the comparative clinical course and outcomes of KD, KD/IgG4-RD, and IgG4-RD. Prospective trials are warranted to determine the optimal treatment method for KD.

## Data availability statement

The original contributions presented in this study are included in the article/supplementary material, further inquiries can be directed to the corresponding author.

## Ethics statement

This study was reviewed approved by the Institutional Review Board of Chang Gung Memorial Hospital (202100959B0 and 202201725B0).

## Author contributions

T-MC: conceptualization, methodology, software, validation, formal analysis, investigation, resources, data curation, visualization, project administration, and funding acquisition. C-CL and T-MC: writing—original draft preparation and writing—review and editing. T-MC and K-HY: supervision. All authors read and agreed to the published version of the manuscript.
